# The willingness and influencing factors to choose smart senior care among old adults in China

**DOI:** 10.1186/s12877-022-03691-3

**Published:** 2022-12-14

**Authors:** Qiyuan Huang, Ying Li, Xiang Wu, Song Ge, Zhe Qu, Aming Wang, Xianping Tang

**Affiliations:** 1 School of Nursing, Xuzhou Medical University, No. 209 Tongshan Road, Xuzhou, 221004 Jiangsu Province China; 2 Department of Emergency, Affiliated Hospital of Xuzhou Medical University, Xuzhou, Jiangsu China; 3 School of Medical Informatics and Engineering, Xuzhou Medical University, Xuzhou, Jiangsu China; 4 Department of Natural Sciences, University of Houston-Downtown, Houston, TX USA; 5 Aging Studies Institute of Xuzhou Medical University, Xuzhou, Jiangsu China

**Keywords:** Smart senior care, Older adults, China; smart device

## Abstract

**Background:**

Population aging has become an escalating issue in China resulting in increasing healthcare demand. Smart senior care has the potential to help older adults live independently and relieve the pressure of healthcare including home-based care. This study aimed to explore Chinese older adults’ preferred access models and service content of smart senior care and factors affecting their willingness to choose smart senior care.

**Methods:**

This was a cross-sectional study. A total of 760 community-dwelling older adults from Xuzhou, China were included in this study. Their demographics, family support, health status, smart senior care use, and willingness to choose smart senior care were collected. The Chi-square test was used for single factor analysis of each variable. The statistically significant variables were included in the logistics regression model to analyze factors influencing older adults’ willingness to choose smart senior care. The chi-square goodness of fit test was used to analyze the preferred content and access models of smart senior care; the Bonferroni method was used to correct the results.

**Results:**

The finding indicated that participants’ age, number of children, frequency of children visiting parents, adequate senior care, self-reported health, chronic diseases, smartphone use, and attitude toward smart senior care were significantly associated with their willingness to choose the smart senior care (*p* < 0.05). For smart senior care access models, participants preferred the remote monitoring model, telephone call model, and the community site model over the health smart home model and the smart application platform model. There was no statistical difference among these three preferred access models (*p’ >* 0.005). Regarding service content, participants desired medical care service the most (*p’* < 0.005).

**Conclusions:**

Chinese older adults’ willingness to choose smart senior care is affected by personal, family, health, and other factors. To develop China’s senior care, we should consider their demand and preference for smart senior care. It is important to enrich the content of smart senior care, especially on medical care services, and maintain the dynamic balance between supply and demand using a diverse supply approach.

**Supplementary Information:**

The online version contains supplementary material available at 10.1186/s12877-022-03691-3.

## Introduction

The increasing proportion of older adults worldwide is a problem now and an inevitable challenge for the future. It poses challenges to economics, people’s livelihood, and social welfare. This issue is particularly prominent in China, a country with improved life expectancy and lowered fertility [1, 2]. China has become an aging society since 1999. In 2021, there were 264 million people aged 60 years or above in China, representing 18.7% of the total population. It is predicted that China will be a super-aged society in 2050, with older adults occupying 34.1% of the total population [3, 4]. With the population aging, the demand for home care and chronic disease management increases [5]. To meet the increasing healthcare demand of older adults in China, the Chinese government has issued the “9073” elderly-care policy and indicated that senior care in China should consist of 90% home care, 7% community care, and 3% institutional care [6]. Therefore, meeting older adults’ home-based senior care needs has become a strategic priority in China.

In recent years, the fourth scientific and technological revolution characterized by the Internet of Things (IoT), Information Technology, Big Data, and Cloud Computing has significantly promoted China’s aging industry [7]. At the same time, a series of policies have been issued to link the internet industry with the aging industry. In this context, the concept of smart senior care emerged and was seriously regarded by the government [8].

Smart senior care refers to the use of information and scientific technology, including health records, medical interventions, and home care to improve the quality of life for older adults [9, 10]. Specifically, through a variety of sensors and network systems remotely monitoring older adults’ daily life, their activities will be recorded and transmitted to healthcare institutions or third-party service companies. Then clinicians can provide online or onsite healthcare interventions including home care assistance [11]. The application of smart senior care has the potential to significantly improve the independence, safety, and quality of life of community-dwelling older adults. This is especially important during the COVID-19 pandemic, when older adults stay home longer and their disconnection from external services increases [12]. Therefore, scientifically developing smart senior care in China is critical.

Through literature review, we found that developed countries have formed relatively large-scale and well-established smart senior care systems [13–15]. In general, their smart senior care models can be divided into five categories: (1) In the remote monitoring model, wearable sensors are used to measure various health parameters of older adults [16, 17]. (2) In the health smart homes model, various forms of assisted living are established to monitor older adults’ daily activities, health status, home environment, assisted mobility, and safety [18, 19]. (3) In the smart application platform or telephone call model, older adults select smart senior services through mobile applications or telephone calls. Then healthcare institutes or third-party service companies can provide online or offline services accordingly [20, 21]. (4) In the community site model, older adults register for services at community stations that provide long-term supervision on disease prevention and emergency treatment [22] (5). In the artificial intelligence model, humanoid robots are used to take care of older adults [23, 24]. All these models allow older adults to live in their familiar environment for as long as possible.

While the development of smart senior care is dominated by technology, evidence emphasizes that it is important to consider older adults’ willingness and demand to choose smart senior care [25]. Lifestyles, health knowledge, cost of services, health status, and safety are likely to be factors influencing the willingness of older adults to choose smart senior care. World Health Organization (WHO) has also proposed to comprehensively consider the dimensions of “health”, “participation”, and “security” to improve the quality of life of older adults and achieve active aging [26]. However, the results of observational and qualitative studies on the influencing factors of older adults’ willingness to choose smart senior care were inconsistent [1, 27, 28]. To the best of our knowledge, there is no cross-sectional study on older adults’ willingness to choose smart senior care in China. Furthermore, two reviews proposed the importance of expanding the content of smart senior care and building a smart senior care access model based on the willingness of older adults [29, 30]. Thus, to promote the development of smart senior care in China, understanding older adults’ willingness to choose smart senior care is essential. Therefore, this study aimed to explore Chinese older adults’ preferred access models and service content of smart senior care and factors affecting their willingness to choose smart senior care. The findings of this study will provide implications for policymakers and providers to better provide smart senior care and meet the growing healthcare demand of older adults in China.

## Methods

### Sampling and inclusion criteria

We used stratified cluster sampling to recruit community-dwelling older adults in Xuzhou, China. Eleven counties and districts in Xuzhou were stratified into three regions based on their economic development and population size. In each region, three communities were randomly selected. The number of distributed questionnaires was proportional to the population size of each selected community. The inclusion criteria were people who (1) were aged 60 or older, (2) were living in communities at the time of the survey; (3) could have rational conversations with researchers. The exclusion criteria were people with cognitive impairment such as dementia or limited vernal ability such as speech disorders. The survey was conducted from June to October 2021. A pilot study was conducted for 1 week prior to the commencement of the study to revise the questionnaire. Two researchers distributed the survey in the communities. To achieve a high response, face-to-face interviews were used to collect the data. A total of 768 participants were recruited, and eight incomplete questionnaires were excluded. Finally, 760 valid questionnaires were collected.

### Questionnaire design

Since there is currently no existing validated survey on older adults’ willingness to choose smart senior care, a structured questionnaire was developed to obtain relevant information. To measure older adults’ willingness to choose smart senior care, the question of “Do you have the willingness to choose smart senior care?” was used. The response was a binary variable (yes or no). Meanwhile, multiple-choice questions were used to investigate participants’ preferred access models and service content of smart senior care. Five categories of smart senior care access models were identified, guided by the current literature [16–22, 27, 28], and were incorporated into the questionnaire, including the remote monitoring model, the health smart homes model, the smart application platform model, the telephone call model, and the community site model. Moreover, we also included questions on five areas of smart senior care service content, including medical care service, home care service, social entertainment service, meal delivery service, and psychological counseling services in the questionnaire.

Factors expected to influence older adults’ willingness to choose smart senior care were summarized in Table 1. In addition to the demographic characteristics of older adults, we added additional independent variables based on the three dimensions of the theory of active aging, including “security”, “health”, and “participation” [26]. 1) Demographic characteristics included age (60–69, 70–79, and 80+), gender (male and female), registered residence (rural and urban), years of education (no education, 1–6 years, and 7+ years), pre-retirement occupation (have a formal job or others). 2) Security dimension included national health insurance enrollment (urban employer medical scheme, urban resident medical scheme, or new rural cooperative medical scheme), monthly income in RMB (< 1000, 1000-1999, 2000-2999, 3000-3999, and more than 4000), live with a spouse (yes or no), number of children (One child, two children, three children, or four or more children), frequency of children visiting parents (every day, weekly, semimonthly, or monthly+), primary caregiver (spouse, children, self, or others), and perceived adequacy of senior care received (yes or no). 3) Health dimension included self-reported health (bad or good), hospitalization within 1 year (yes or no), any chronic diseases (yes or no), frequency of physical examination (half a year, annually, biennial, or more than 2 years), and any disability in instrumental activities of daily living (IADLs) (yes or no). IADLs were measured based on eight self-reported activities: (a) visiting neighbors, (b) shopping, (c) cooking, (d) washing clothes, (e) walking 1 kilometer, (f) lifting 5 kg, (g) crouching, and standing up three times, and (h) taking public transportation. 4) Participation dimension covered the key factors of older adults’ participation in smart senior care, included smartphone use (yes or no), familiarity with smart senior care (know very well, heard of, or never heard of), and attitude towards smart senior care (supportive or not). Smartphone was defined as a type of mobile phone with an independent operating system and running space where users can install applications developed by third-party service providers and achieve wireless network access through mobile communication networks [31].

**Table 1:** Categorization of Variables

Variable name	Categorization
Demographic characteristics	
Age	Aged 60–69 = 0, Aged 70–79 = 1, or Aged 80^+^ = 2
Gender	Female = 0, male = 1
Registered residence	Rural = 0, urban = 1
Years of education	No education = 0, 1–6 years = 1, or 7 or more years of education = 2
Pre-retirement occupation	A dichotomized variable - 1 if the participants had a formal job before retirement, 0 if not.
Security factors	
National health insurance enrollment	Enrolled in Urban Employer Medical Scheme, UEMS = 0, Enrolled in Urban Resident Medical Scheme, URMS = 1, Enrolled in New Rural Cooperative Medical System, NRCMS = 2
Monthly income (RMB)	Less than 1000 = 0, 1000–1999 = 1, 2000–2999 = 2, 3000–3999 = 3, or more than 4000 = 4
Live with a spouse	A dichotomized variable - 1 if the participants’ have living spouse, 0 if not
Number of children	One child or 0 = 0, two children = 1, three children = 2, and four or more children = 3
Frequency of children visiting parents	Everyday = 0, weekly = 1, semimonthly = 2, monthly^+^ = 3
Primary caregiver	Spouse = 0, children = 1, oneself = 2, and others = 3
Perceived adequacy of senior care received	A dichotomized variable - 1 if the participants could receive adequate senior care, 0 if not
Health factors	
Self-reported health	A dichotomized variable - well = 0 and worse = 1
Any IADL disability	A dichotomized variable −1 if the participants needed any help performing the following tasks: visiting neighbors, washing clothes, walking 1 kilometer, shopping, cooking, lifting 5 kg, crouching, and standing up three times, and using public transportation, 0 if not
Hospitalization within a year	Yes = 1, no = 0
Any chronic diseases	A dichotomized variable - 1 if the participants reported hypertension, heart diseases, cerebrovascular diseases, or other diseases, 0 if not
Frequency of physical examination	Half a year = 0, annually = 1, biennial = 2, and more than 2 years = 3
Participation factors	
Smartphone use	A dichotomized variable - 1 if the participants used smartphone, 0 if not
Familiarity with smart senior care	A metataxonomic variable, according to the participants’ stated knowledge about smart senior care. Know very well = 0, just heard of = 1, incomprehension = 2
Attitude towards smart senior care	A dichotomized variable - 1 if the participants were supportive of smart senior care, 0 if not

### Procedure and statistical analysis

The study proposal was ethically reviewed and approved by the Human-related Research Ethical Committee of the Affiliated Hospital of Xuzhou Medical University (XYFY2021-KL157–01). When consenting participants for enrollment, we explained the purpose of the study to them. The confidentiality of data storage was also emphasized, and their written informed consent was obtained. After the questionnaire was returned to us, we confirmed on site if there were any questions that participants did not understand and checked with them. Two researchers imported the data into SPSS 25.0 statistical software. Missing values for a small number of questionnaires were handled by multiple imputations by SPSS 25.0 software.

The Chi-square test was used to examine the correlations between the independent variables and dependent variable. Then, logistic regression was constructed to analyze factors affecting older adults’ willingness to choose smart senior care. The Hosmer-Lemeshow test was applied to assess the goodness of fit of the logistic regression model [32]. The null hypothesis H0 (the model provides a good fit) and alternative hypothesis H1 (the model does not fit the data) were tested, respectively. We also performed variance inflation factor (VIF) calculations of the independent variables to determine whether multicollinearity existed among the variables. Finally, we used count data to calculate participants’ preferences on the smart senior care access models and service content. The chi-square goodness of fit test was used to test whether there were statistical differences among different groups. Due to the increased risk of a type I error when making multiple statistical tests, the Bonferroni correction method was adopted when we examined the difference in probability (*p’*) values of multiple groups [33, 34]. All statistical tests were two-sided, with *p* < 0.05 considered statistically significant.

## Results

### Characteristics of the participants

Participants’ characteristics were reported in Table 2. Most participants aged between 70 and 79 (41.7%) were females (51.2%), rural residents (58.3%), and did not have a formal job before retirement (55.4%). The participants were poorly educated- only 32.4% had over 7 years of education. On the type of medical insurance, most participants (42.2%) used the new rural cooperative medical system (NRCMS). Most participants (30.1%) had a monthly income between 2000 and 2999 RMB (about 292.61 ~ 438.76 USD), and 21.4% of the participants had a monthly income of less than 1000 RMB (about 146.30 USD).

**Table 2:** Characteristics of the Participants

Variables	Total (*n* = 760)	If willing to choose smart senior care		*χ*^*2*^
		Yes	No	
Demographic characteristics				
Age, %				
Ages 60–69	275 (36.2)	235 (85.5)	40, (14.5)	38.248^***^
Ages 70–79	317 (41.7)	257 (81.1)	60 (18.9)	
Ages 80^+^	168 (22.1)	103 (61.3)	65 (38.7)	
Gender, %				
Female	389 (51.2)	301 (77.4)	88 (22.6)	0.390
Male	371 (48.8)	294 (79.2)	77 (20.8)	
Registered residence, %				
Rural	443 (58.3)	332 (74.9)	111 (25.1)	6.995^**^
Urban	317 (41.7)	263 (83.0)	54 (17.0)	
Years of education, %				
No education	133 (17.5)	89 (67.0)	44 (33.0)	24.283^***^
1–6 years of education	381 (50.1)	290 (76.1)	91 (23.9)	
7 + years of education	246 (32.4)	216 (87.8)	30 (22.2)	
Pre-retirement occupation, %				
Have a formal job	339 (44.6)	278 (82.0)	61 (18.0)	4.973^*^
Others	421 (55.4)	317 (75.3)	104 (24.7)	
Security factors				
National health insurance enrollment, %				
UEMS	256 (33.7)	215 (84.0)	41 (16.0)	9.197^*^
URMS	183 (24.1)	144 (78.7)	39 (21.3)	
NRCMS	321 (42.2)	236 (73.5)	85 (26.5)	
Monthly income in RMB, %				
Less than 1000	163 (21.4)	121 (74.2)	42 (25.8)	11.350^*^
1000-1999	150 (19.7)	109 (72.7)	41 (27.3)	
2000-2999	229 (30.1)	179 (78.2)	50 (21.8)	
3000-3999	132 (17.4)	115 (87.1)	17 (12.9)	
More than 4000	86 (11.3)	71 (82.6)	15 (17.4)	
Live with a spouse, %				
Yes	605 (79.6)	475 (78.5)	130 (21.5)	0.087
No	155 (20.4)	120 (77.4)	35 (22.6)	
Number of children, %				
Only child	192 (25.3)	178 (92.7)	14 (7.3)	43.073^***^
Two children	278 (36.6)	214 (77.0)	64 (23.0)	
Three children	162 (21.3)	123 (76.0)	39 (24.0)	
Four or more children	128 (16.8)	80 (62.5)	48 (37.5)	
Frequency of children visiting parents, %				
Everyday	258 (33.9)	180 (69.8)	78 (30.2)	25.384^***^
Weekly	242 (31.8)	192 (79.3)	50 (20.7)	
Semimonthly	107 (14.1)	84 (78.5)	23 (21.5)	
Monthly^+^	153 (20.2)	139 (90.8)	14 (9.2)	
Primary caregiver, %				
Spouse	276 (36.3)	227 (82.2)	49 (17.8)	4.632
Children	219 (28.8)	164 (74.9)	55 (25.1)	
Oneself	243 (32.0)	186 (76.5)	57 (23.5)	
Others	22 (2.9)	18 (81.8)	4 (18.2)	
Perceived adequacy of senior care received, %				
Yes	263 (34.6)	185 (70.3)	78 (29.7)	14.944^***^
No	497 (65.4)	410 (82.5)	87 (17.5)	
Health factors				
Self-reported health, %				
Well	459 (60.4)	340 (74.1)	119 (25.9)	12.116^***^
Worse	301 (39.6)	255 (84.7)	46 (15.3)	
Any IADL disability, %				
Yes	492 (64.7)	393 (79.9)	99 (20.1)	2.072
No	268 (35.3)	202 (75.4)	66 (24.6)	
Hospitalization within a year, %				
Yes	231 (30.4)	194 (84.0)	37 (16.0)	6.329^*^
No	529 (69.6)	401 (75.8)	128 (24.2)	
Any chronic diseases, %				
Yes	426 (56.1)	350 (82.2)	76 (17.8)	8.542^**^
No	334 (43.9)	245 (73.3)	89 (26.7)	
Frequency of physical examination, %				
Half a year	157 (20.7)	125 (79.6)	32 (20.4)	19.977^***^
Annually	325 (42.8)	270 (83.1)	55 (16.9)	
Biennial	129 (17.0)	103 (79.8)	26 (20.2)	
More than 2 years	149 (19.6)	97 (65.1)	52 (34.9)	
Participation factors				
Smartphone use, %				
Yes	441 (58.0)	371 (84.1)	70 (15.9)	21.064^***^
No	319 (42.0)	224 (70.2)	95 (29.8)	
Familiarity with smart senior care, %				
Know very well	48 (6.3)	41 (85.4)	7 (14.6)	6.453^*^
Just heard of	227 (29.9)	188 (82.8)	39 (17.2)	
Incomprehension	485 (63.8)	366 (75.5)	119 (24.5)	
Attitude towards smart senior care, %				
Supportive	686 (90.3)	573 (83.5)	113 (16.5)	113.737^***^
Not supportive	74 (9.7)	22 (29.7)	52 (70.3)	

*** *p* < 0.001, ** 0.001 ≤ *p* < 0.01, * 0.01 ≤ *p* < 0.05

Regarding family support, most participants lived with a spouse (79.6%). One-third of the participants’ primary caregivers (36.3%) were their spouses, followed by the participants themselves (32.0%). One-third of the participants (36.6%) had two children, and 33.9% could see their children daily. Over half of the participants could not get adequate senior care (65.4%). In addition, most participants (60.4%) were in good health. One-third of the participants (30.4%) were hospitalized in the past year. More than half of the participants (56.1%) had chronic diseases or any IADL disability (64.7%). About half of the participants (42.8%) had an annual physical examination.

Regarding the participation dimension, more than half of the participants (58.0%) used a smartphone. After the researcher explained the purpose of the study and the content of smart senior care, 90.3% of the participants were willing to choose smart senior care. However, many participants (63.8%) had never heard of smart senior care before this study.

### The logistic regression analysis result

Table 2 presented the results of correlation analysis between the independent variables and dependent variable. We found that age, registered residence, years of education, pre-retirement occupation, type of national health insurance enrollment, monthly income, number of children, frequency of children visiting parents, perceived adequacy of senior care received, self-reported health, hospitalization within a year, any chronic diseases, physical examination frequency, smartphone use, familiarity with smart senior care, and attitude towards smart senior care were significantly related the participants’ willingness to choose smart senior care. We included these statistically significant correlated variables in the logistic regression model. The result displayed that the *p*-value of the Hosmer-Lemeshow test exceeded 0.05 for the models. Thus, this binary logistic regression model fits well with no statistical deviation between the agreement fitting model and the actual model [35]. And the VIF values were all less than 10. Thus, there was no multicollinearity among the independent variables.

The logistic regression results (Table 3) revealed that participants’ age, number of children, frequency of children visiting parents, perceived adequacy of senior care, self-reported health, chronic diseases, smartphone use, and attitude towards smart senior care were found to be significantly associated with the participants’ willingness to choose the smart senior care (*p* < 0.05). Specifically, the participants who were over 80 years old (OR = 0.457), had more than one child, had more child visiting time, and received adequate senior care (OR = 0.569) were less willing to choose smart senior care than the intra-group reference people. Moreover, the participants who had worse self-reported health (OR = 2.332), lived with chronic diseases (OR = 2.378), used smartphones (OR = 1.674), and were supportive of smart senior care (OR = 13.493) were more willing to choose smart senior care than the intra-group reference people.

**Table 3:** Factors including the participants’ willingness to choose smart senior care

Factors	*β*	S. E	Wald	*p*-value	Exp(B)	95%*CI*	VIF
Age (Reference: age 60–69)							1.476
Age 70–79	−0.016	0.293	0.003	0.957	0.984	0.554–01.748	
Age 80^+^	**−0.793**	**0.337**	**5.412**	**0.020**	**0.457**	**0.236–0.884**	
Registered residence (Reference: rural)							1.997
Urban	0.154	0.313	0.243	0.622	1.167	0.632–2.153	
Years of education (Reference: no education)							1.848
1–6 years of education	−0.193	0.297	0.421	0.517	0.825	0.461–1.476	
> 6 years of education	0.140	0.426	0.108	0.742	1.150	0.499–2.653	
Pre-retirement occupation (Reference: Have a formal job)							2.333
Others	0.493	0.369	1.787	0.181	1.638	0.795–3.376	
National health insurance enrollment (Reference: UEMS)							3.644
URMS	−0.299	0.420	0.504	0.478	0.742	0.325–1.691	
NRCMS	−0.513	0.482	1.136	0.286	0.598	0.233–1.538	
Monthly income in RMB (Reference: less than 1000)							2.465
1000-1999	−0.601	0.340	3.121	0.077	0.548	0.282–1.068	
2000-2999	−0.464	0.355	1.715	0.190	0.628	0.314–1.259	
3000-3999	0.269	0.494	0.297	0.586	1.309	0.497–3.443	
More than 4000	−0.425	0.577	0.543	0.461	0.654	0.211–2.025	
Number of children (Reference: only one child)							1.393
Two children	**−1.115**	**0.357**	**9.776**	**0.002**	**0.328**	**0.163–0.660**	
Three children	**−1.083**	**0.399**	**7.387**	**0.007**	**0.339**	**0.155–0.739**	
Four or more children	**−1.481**	**0.417**	**12.611**	**0.001**	**0.227**	**0.100–0.515**	
Frequency of children visiting parents (Reference: everyday)							1.104
Weekly	0.362	0.262	1.917	0.166	1.436	0.860–2.398	
Semimonthly	0.115	0.342	0.113	0.737	1.122	0.574–2.192	
Monthly^+^	**1.340**	**0.379**	**12.506**	**0.001**	**3.818**	**1.817–8.021**	
Perceived adequacy of senior care received (Reference: no)							1.259
Yes	**−0.563**	**0.238**	**5.610**	**0.018**	**0.569**	**0.357–0.907**	
Self-reported health (Reference: Well)							1.361
Worse	**0.847**	**0.280**	**9.126**	**0.003**	**2.332**	**1.346–4.040**	
Hospitalization within a year (Reference: no)							1.308
Yes	0.168	0.285	0.349	0.555	0.845	0.483–1.477	
Any chronic diseases (Reference: No)							1.521
Yes	**0.857**	**0.402**	**9.704**	**0.010**	**2.378**	**1.208–4.490**	
Frequency of physical examination (Reference: half a year)							1.264
Annually	0.109	0.311	0.122	0.727	1.115	0.605–2.053	
Biennial	−0.192	0.365	0.275	0.600	0.826	0.404–1.689	
More than 2 years	−0.431	0.366	1.389	0.239	0.650	0.317–1.331	
Smartphone use (Reference: No)							1.383
Yes	**0.515**	**0.247**	**4.358**	**0.037**	**1.674**	**1.032–2.717**	
Familiarity with smart senior care (Reference: know very well)							1.334
Just heard of	0.233	0.482	0.234	0.629	1.102	0.308–2.036	
Incomprehension	0.112	0.581	0.037	0.847	1.119	0.358–3.493	
Attitude towards smart senior care (Reference: not supportive)							1.051
Supportive	**2.602**	**0.330**	**32.046**	**0.000**	**13.493**	**7.062–25.781**	

Note. Covariates were calculated by the minimum assignment.*β* standardized regression coefficient, *S.E* standard error, *VIF* variance inflation factor

### The chi-square goodness of fit test results

**Fig. 1:**
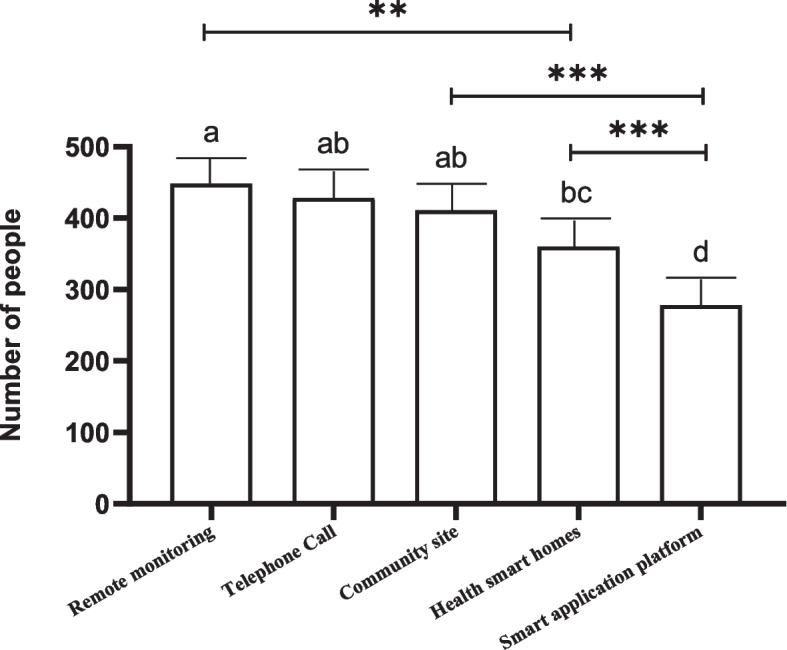
Comparison the differences of the five access models selected by the participants. If the groups contain the same alphabet, there is no statistical difference between/among them. *** *p’* < 0.001, ** 0.001 ≤ *p’* < 0.005

Additional file 1: Supplement Table 1 showed the chi-square goodness of fit test results of the five smart senior care access models. The results showed that the remote monitoring model was the most popular access model among the participants (selected by 448 participants). The least popular access model was the smart application platform model (selected by 278 participants). After Bonferroni correction, we found that the remote monitoring model, the telephone call model, and the community site model were more popular among participants than the other two models. In addition, there was no statistical difference among the three access models. The health smart homes model had a statistical difference from the remote monitoring model. Still, there was no statistical difference between the health smart homes model, the telephone call model, and the community site model. The smart application platform model was the least selected and statistically different from other access models. Figure 1 showed a comparison of the participants’ willingness to choose the five access models.

**Fig. 2:**
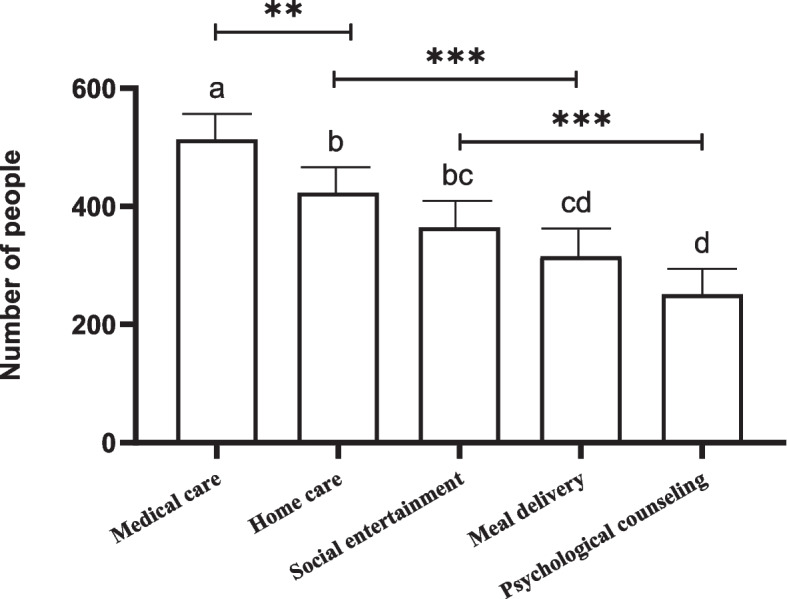
Comparison the differences of the five service content selected by the participants. If the groups contain the same alphabet, there is no statistical difference between them. *** *p’* < 0.001, ** 0.001 ≤ *p’* < 0.005

Additional file 1: Supplement Table 2 showed the chi-square goodness of fit for the five smart senior care service types. After Bonferroni correction, we found that medical care services were the most popular service content (selected by 513 participants) with a statistical difference from other types of service content. Four hundred twenty-three participants chose home care services; 364 chose social entertainment services, and there was no statistical difference between them. Psychological counseling service was the least popular (selected by 251 participants), and there was no statistical difference between it and meal delivery service. Figure 2 showed a comparison of the participants’ willingness to choose the five service contents.

## Discussion

To the best of our knowledge, there are few cross-sectional study examining older adults’ demands and willingness to choose smart senior care in China. In this study, we found that the health of older adults in Xuzhou, China was poor, and their quality of life was not high. Our finding on older adults’ health status was dissimilar to the findings of the Chinese Longitudinal Healthy Longevity Survey (CLHLS) [36, 37]. While 56.1% of the participants suffered from chronic diseases in our study, the latter was 60.53%. While more than 60% had varying degrees of IADL disorders in our study, the latter was 49.91%. One reason for this discrepancy may be that our participants had a more advanced age than those in the Chinese Longitudinal Healthy Longevity Survey [38]. The family structure change, the anticipated increasing burden of chronic disease, and the possible attenuation of filial care increased demand for home-based care in China. At the same time, we found that the demand for information services for older adults was growing. 58.0% of the participants used smartphones, which is higher than what the China Internet Network Information Center (CNNIC) reported in December 2021 (43.2%) [39]. The difference may be due to the different samples in the two studies. In addition, as reported in the CNNIC, 80.8% of older adults who use mobile phones will actively seek Internet lifestyle care services [39]. And in this study, 90.3% of participants supported smart senior care. This condition created an excellent opportunity for developing smart senior care in China.

In terms of the participants’ willingness to choose smart senior care, we found that older adults’ willingness to choose smart senior care was affected by many aspects. Firstly, compared with younger older adults, the participants over 80 years old had less willingness to choose smart senior care. This may be due to the influence of traditional culture and their limited ability to accept new things, resulting in their lowered willingness to choose smart senior care. Thus, companies should consider the older adults’ age when promoting smart senior care. Compared with older adults with advanced age, younger older adults are more likely to accept smart senior care, are the main consumers of smart senior care, and have more demands for smart senior care.

Secondly, regarding the living security dimension, the number of children, frequency of children visiting parents, and perceived adequacy of senior care received influenced participants’ willingness to choose smart senior care. Specifically, the participants with only one child, who were visited less frequently by their children, and did not receive adequate senior care at home were more willing to choose smart senior care than those with more than one child. Gordana Dermody et al. have also indicated that family support affects older adults’ willingness to choose smart senior care. Older adults with less family support were more likely to receive smart senior care [27]. It is not hard to tell that the “4 + 2 + 1″ family model (A family unit that consists of four old adults, two adults, and one child) due to the one-child policy poses severe challenges to China’s current home care situation [40, 41]. Older adults who did not receive adequate living security were more willing to choose smart senior care. Therefore, when implementing smart senior care, we should pay special attention to older adults who live alone or have empty nesters. In addition, the government should develop customized technology solutions for older adults with low living security to meet their healthcare need.

Thirdly, the participants with poor self-reported health or chronic conditions were more willing to choose smart senior care. In another study in South Korea, researchers reported that older adults’ health was related to their willingness to choose smart senior care [1]. Older adults have paid more attention to their health with improved living standards. Those with poor physical conditions have become willing to promote their health through the new healthcare model. Thus, we should develop telemedicine to meet older adults’ healthcare demands. The government could also consider incorporating smart senior care into residents’ medical insurance to reduce the burden of family health care.

Fourthly, some factors in the participation dimension also affect older adults’ willingness to choose smart senior care. The results showed that the participants who have used smartphones were more willing to choose smart senior care. Yuanyuan Cao et al. also reported that older adults with rich experience using smart products were more likely to choose smart senior care [28]. Thus, improving older adults’ information technology levels is another crucial task at present. We can try to let older adults experience the convenience of smart senior care. In addition, through digital training or intelligent experience, we can help them better use smart products.

Concerning the smart senior care access models, we found that older adults preferred the remote monitoring, telephone call, and community site access models over the smart application platform model or health smart homes model. On the one hand, it is related to the fact that participants could not use smart products or were not proficient in using them. On the other hand, older adults may worry about their limited learning ability, resulting in poor, smart product operation. Nthubu Badziili has also indicated that more consideration should be given to the interaction among users, sensors, and data when integrating smart products into the home environment of older adults [30]. Therefore, to develop smart senior care in China, we must overcome the “digital divide” problem among older adults. Companies offering smart senior care should implement customer-oriented strategies and take the initiative to achieve user-friendliness for older adults. Eventually, we hope to promote the popularity of smart senior care and benefit older adults by optimizing their access to smart senior care.

On participants’ willingness for service content, we found that old adults’ smart senior care willingness for medical care services was highlighted. Majumder, Sumit et al. also pointed out the importance of medical care services in smart senior care [29]. Therefore, smart senior care should take advantage of information technology to ensure the supply of medical care service resources. Combined with the analysis results of the smart senior care access model, we can collect dynamic data such as vital signs by wearing wearable devices on older adults’ bodies and then monitor their physical state remotely. Of course, if we need to establish smart senior care base stations in communities, relevant staff should manage health records and track the life trajectories of older adults at home in the region. In addition, alarm-calling devices should be installed at home to facilitate old adults in need of timely contact with healthcare institutions. Through a series of measures, we will find a suitable way to develop smart senior care in China so that more old adults can understand and accept smart senior care.

## Limitations

This study has some limitations. To start with, the sample size of this study is relatively small. Only 760 older adults were included in the study, and all were from Xuzhou, China. Thus, our study has limited generalizability. In addition, we acknowledge that some variables, such as health status, were assessed using self-report. Future studies are needed to expand the scope of the survey to make the study population more representative and use validated questionnaires to assess the variables.

## Conclusions

The findings of this study indicate that older adults’ willingness to choose smart senior services was affected by their personal, family, health, and other factors. Thus, we should consider the characteristics of older adults and provide them with tailored smart senior care. At the same time, we should adopt diverse supply methods, expand service content, and utilize the latest technologies, such as the Internet of Things, big data, and cloud computing, to introduce smart senior care to older adults and their family members. With the support and cooperation of all sectors of society, we hope to gradually establish smart senior care suitable for China.

## Supplementary Information

**Additional file 1.**

## Data Availability

The data that support the findings of this study are available on request from the corresponding author. The data are not publicly available due to privacy or ethical restrictions.

## Abbreviations

IoT: Internet of Things
WHO: World Health Organization
IADL: Instrumental activities of daily living
UEMS: Urban Employer Medical Scheme
URMS: Urban Resident Medical Scheme
NRCMS: New Rural Cooperative Medical System
CLHLS: Chinese Longitudinal Healthy Longevity Survey
CNNIC: China Internet Network Information Center
