# Supplementary material for: The willingness and influencing factors to choose smart senior care among old adults in China
Source: BMC Geriatr. 2022 Dec 14;22:967. doi: 10.1186/s12877-022-03691-3 (PMC9750727; doi:10.1186/s12877-022-03691-3)
Supplement: Supplementary file 1 — Additional file 1. [file 12877_2022_3691_MOESM1_ESM.docx]

**Supplement Table 1** The Chi-square goodness of fit test results of the five access models

| Category | Total  (n = 760) | Model 1 | Model 2 | Model 3 | Model 4 | Model 5 |
| --- | --- | --- | --- | --- | --- | --- |
| 1. The remote monitoring model | 448 | 1 |  |  |  |  |
| 2.The telephone call model | 428 | 0.457 | 1 |  |  |  |
| 3. The community site model | 411 | 1.594 | 0.344 | 1 |  |  |
| 4. The health smart homes model | 360 | **12.831^***^** | 5.868 | 3.374 | 1 |  |
| 5. The smart application platform model | 278 | **39.807^***^** | **31.870^***^** | **25.673^***^** | **10.539^**^** | 1 |

Note. *** *p**’*< 0.001, ** 0.001 ≤ *p’*< 0.005 Chi-square goodness of fit test was performed for 10 times, and the test level after Bonferroni correction was α’ = 0.005.

**Supplement Table 2** The Chi-square goodness of fit test results of the five service contents

| Category | Total  (n = 760) | Service 1 | Service 2 | Service 3 | Service 4 | Service 5 |
| --- | --- | --- | --- | --- | --- | --- |
| 1.Medical care service | 513 | 1 |  |  |  |  |
| 2.Home care service | 423 | **8.654^**^** | 1 |  |  |  |
| 3.Social entertainment service | 364 | **25.315^***^** | 4.423 | 1 |  |  |
| 4.Meal delivery service | 315 | **47.348^***^** | **15.805^***^** | 3.536 | 1 |  |
| 5.Psychological counseling service | 251 | **89.848^***^** | **43.893^***^** | **20.763^***^** | 7.237 | 1 |

Note. *** *p’*< 0.001, ** 0.001 ≤ *p’*< 0.005 , α’ = 0.005.
